# Phyllodes Tumor of the Breast Enlarged by Hemorrhagic Infarction: A Case Report

**DOI:** 10.7759/cureus.72073

**Published:** 2024-10-21

**Authors:** Yoshika Nagata, Yui Yamada, Izumi Kinoshita, Toshihiro Saeki, Takahisa Fujikawa

**Affiliations:** 1 Surgery, Kokura Memorial Hospital, Kitakyushu, JPN; 2 Pathology, Kokura Memorial Hospital, Kitakyushu, JPN

**Keywords:** breast tumor, hemorrhagic infarction, pathological diagnosis, phyllodes tumor, surgical intervention

## Abstract

Breast phyllodes tumors are rare mesenchymal tumors, often accompanied by internal cysts, hemorrhages, infarctions, and necrosis. The tumor exhibits rapid growth, especially when infarct necrosis occurs within the tumor. In the current report, we showed a woman in her 50s who noticed a rapidly growing breast mass and received an excisional biopsy diagnosis of a phyllodes tumor with hemorrhagic infarction. Pathological diagnosis was challenging due to severe hemorrhage and necrosis; however, a comprehensive diagnosis that considered imaging tests recommended appropriate surgical treatment. Here, we report our experience and further analyze the existing literature regarding the prognostic impact of phyllodes tumors enhanced by intratumoral changes.

## Introduction

Phyllodes tumors are rare fibroepithelial neoplasms that arise from the mesenchymal tissue of the breast, accounting for less than 1% of all breast tumors [1]. These tumors are classified pathologically as benign, borderline, or malignant [2,3]. A higher proliferative potential correlates with an increased risk of local recurrence and metastasis. Therefore, surgery is frequently required for both diagnostic and therapeutic purposes when a phyllodes tumor is suspected.

As clinicians strive to refine treatment strategies, understanding the intricate intratumoral dynamics becomes paramount. Phyllodes tumor growth may exhibit findings including hemorrhage, necrosis, and cystic degeneration. Intratumoral hemorrhage and infarction progress to intratumoral necrosis. Hemorrhagic infarction resulting in necrosis frequently causes rapid tumor enlargement. While these tumors exhibit various histological features, the impact of specific intratumoral events such as infarction, hemorrhage, and necrosis on prognosis remains underexplored.

We herein report a case of an enlarged phyllodes tumor of the breast caused by hemorrhagic infarction and aim to shed light on the prognostic significance of intratumoral changes within phyllodes tumors.

## Case presentation

A woman in her 50s from the Philippines observed an abrupt, painful mass in her right breast during the period of movement restrictions due to the COVID-19 pandemic. The pain subsided spontaneously, and there was no subsequent enlargement, therefore she monitored her condition herself and came to our outpatient clinic two years later without getting infected with COVID-19. Her past medical history is unremarkable, and her family history includes an aunt with breast cancer. Her blood pressure is within a normal range, and there are no oral medications, such as anticoagulants. The physical examination revealed an elastic hard lump with a relatively smooth surface and irregular margins located in the inner upper quadrant of the right breast. No skin changes or dimpling were observed. There were no inflammatory signs and no palpable lymphadenopathy. Blood tests showed that total cholesterol and triglycerides were 217 mg/dl (reference range: 130-219 mg/dL) and 52 mg/dl (reference range: 30-149 mg/dL), respectively. All laboratory data, including coagulation capacity, were normal; however, positive results for hepatitis B virus were detected. Her viral load was 3.3 Log IU/ml, which is above the normal range. There were no signs of chronic hepatitis, and an abdominal ultrasound revealed a fatty liver without any abnormal findings like cirrhosis.

**Figure 1:**
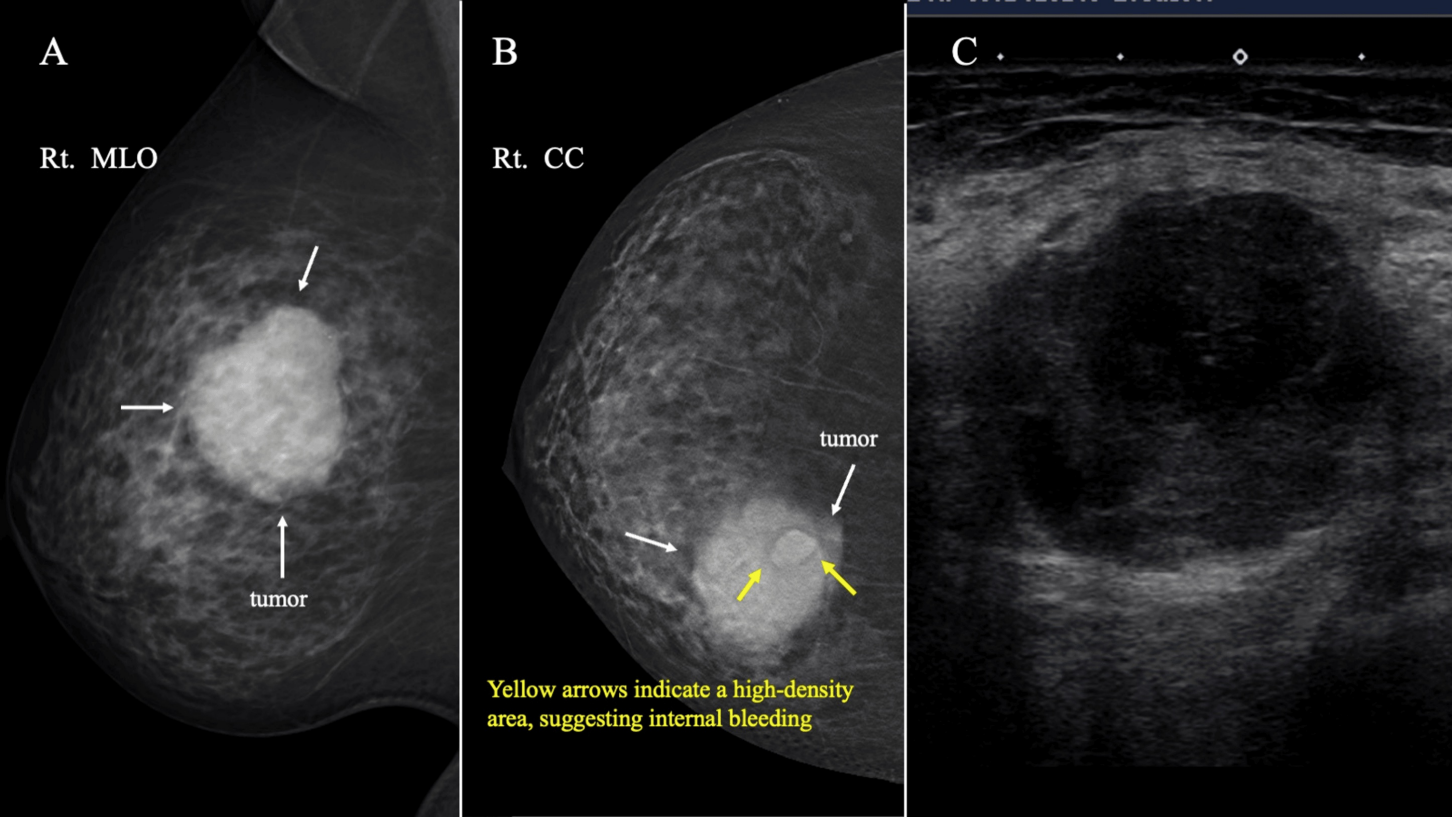
MG and US findings. (A) MG shows a lobulated, high-density mass (white arrow) on medio-lateral oblique view. (B) A craniocaudal view of 3D-MG showed a lobulated mass (white arrow) with relatively high-density area (yellow arrow) on the internal chest wall side of the tumor. The yellow arrows indicate a high-density area, suggesting internal bleeding. (C) US findings. US revealed a hypoechoic, heterogeneous tumor with an unclear border. MG: Mammography, US: Ultrasonography, 3D-MG: Tomosynthesis.

Mammography (MG) showed a well-defined, round, partially lobulate-shaped mass in the upper inner regions of the right breast. Tomosynthesis (3D-MG) showed a relatively high-density area on the inner chest wall side of the tumor. An MG and ultrasonography (US) taken 10 years ago at a breast cancer screening showed no abnormalities. US indicated a low echoic tumor with partially irregular borders measuring 4.3 cm in diameter. The tumor exhibited heterogeneous and hypoechoic echo levels, with no observed blood flow signal (Figure 1). US detected an enlarged axillary lymph node with diffuse cortical thickening, which was diagnosed as reactive lymphadenopathy based on its shape and bilaterality.

**Figure 2:**
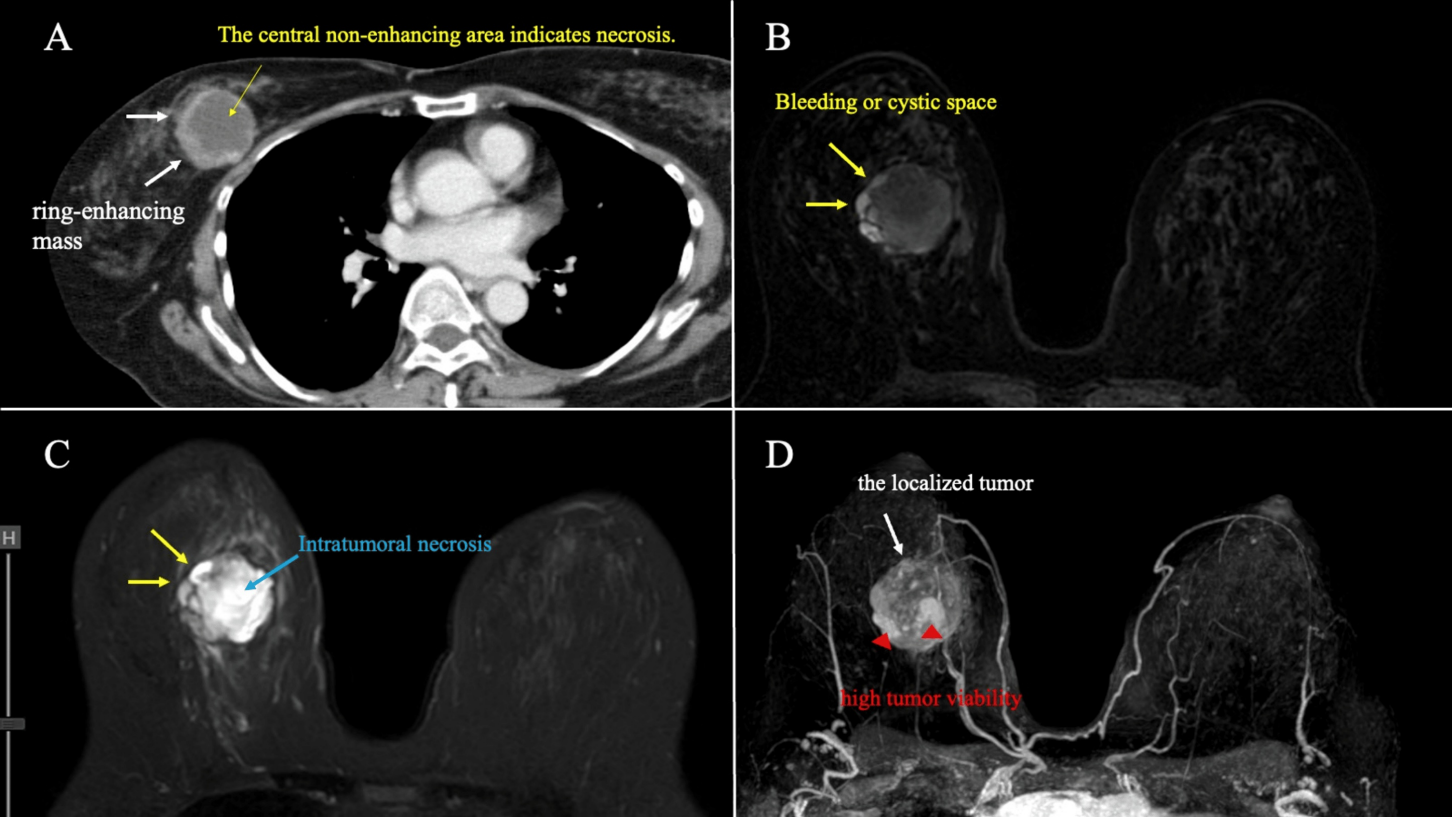
Contrast-enhanced CT and MRI findings. (A) CT showed a ring-enhancing mass (white arrow). (B, C) The tumor margins exhibited elevated SI on both T1WI and T2WI in MRI (yellow arrow). The yellow arrows indicating internal bleeding or cystic regions. (D) MIP images show the localized tumor (white arrow) with no obvious intraductal spread. Tumor showed areas of high proliferative potential (red arrowheads). CT: Computed tomography, MRI: Magnetic resonance imaging, SI: signal intensity, WI: weighted images, MIP: Maximum intensity projection.

Computed tomography (CT) showed strongly enhanced tumors with contrast enhancement at the margins of the mass, while the center of the tumor was not stained. There was no significant distant metastasis. Magnetic resonance imaging (MRI) revealed a lobulated-shaped tumor with an irregular margin, displaying a low signal intensity (SI) on T1-weighted images (WI) and a high SI on T2WI with a low enhancement effect in the center of the tumor. The dynamic study shows a medium persistent pattern on the time intensity curve (TIC) in the middle of the tumor. The tumor margins, which are internal high-density areas of MG, showed high SI on both T1WI and T2WI in MRI. The part of the tumor showed high intensity staining in the ultrafast phase in dynamic studies and indicated the high tumor viability. These imaging results indicated that the interior of the cystic mass was necrotic and bleeding. Maximum intensity projection (MIP) images show that the tumor was relatively localized and showed internal heterogeneous enhancement, with no obvious intraductal extension (Figure 2).

**Figure 3:**
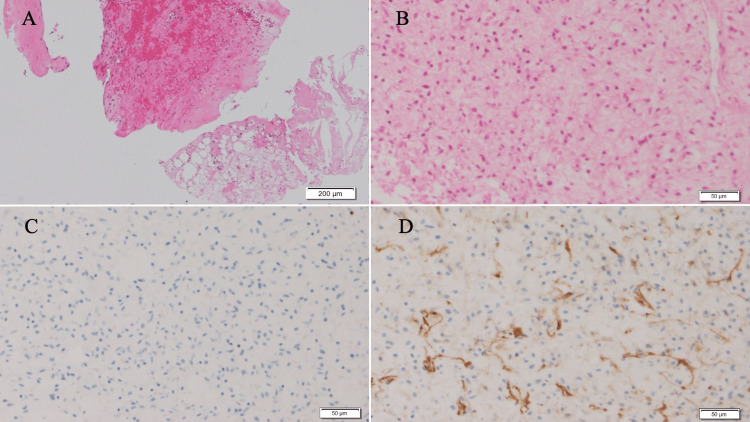
Pathological findings of CNB. The fragmented breast tissue was stained with hematoxylin and eosin staining, which revealed (A) fibroedematous stroma with hemorrhage (scale bar: 200 μm) and (B) short spindle cells (scale bar: 50 μm). Immunohistological staining of the spindle cells showed (C) S-100 (-), (D) CD34 (+, focal) (scale bar: 50 μm). CNB: Core needle biopsy, CD: Cluster of differentiation.

Core needle biopsy (CNB) showed fibroedematous stroma with hemorrhage infiltration of neutrophils and lymphocytes, and hyperplasia of short spindle cells. The immunohistological staining of the spindle cells revealed a negative result for S-100 and a positive result for Cluster of differentiation 34 (CD34), with focal distribution (Figure 3). There were no obvious malignant findings, and the tumor was diagnosed as a benign tumor, such as reactive tissue with coagulation necrosis.

**Figure 4:**
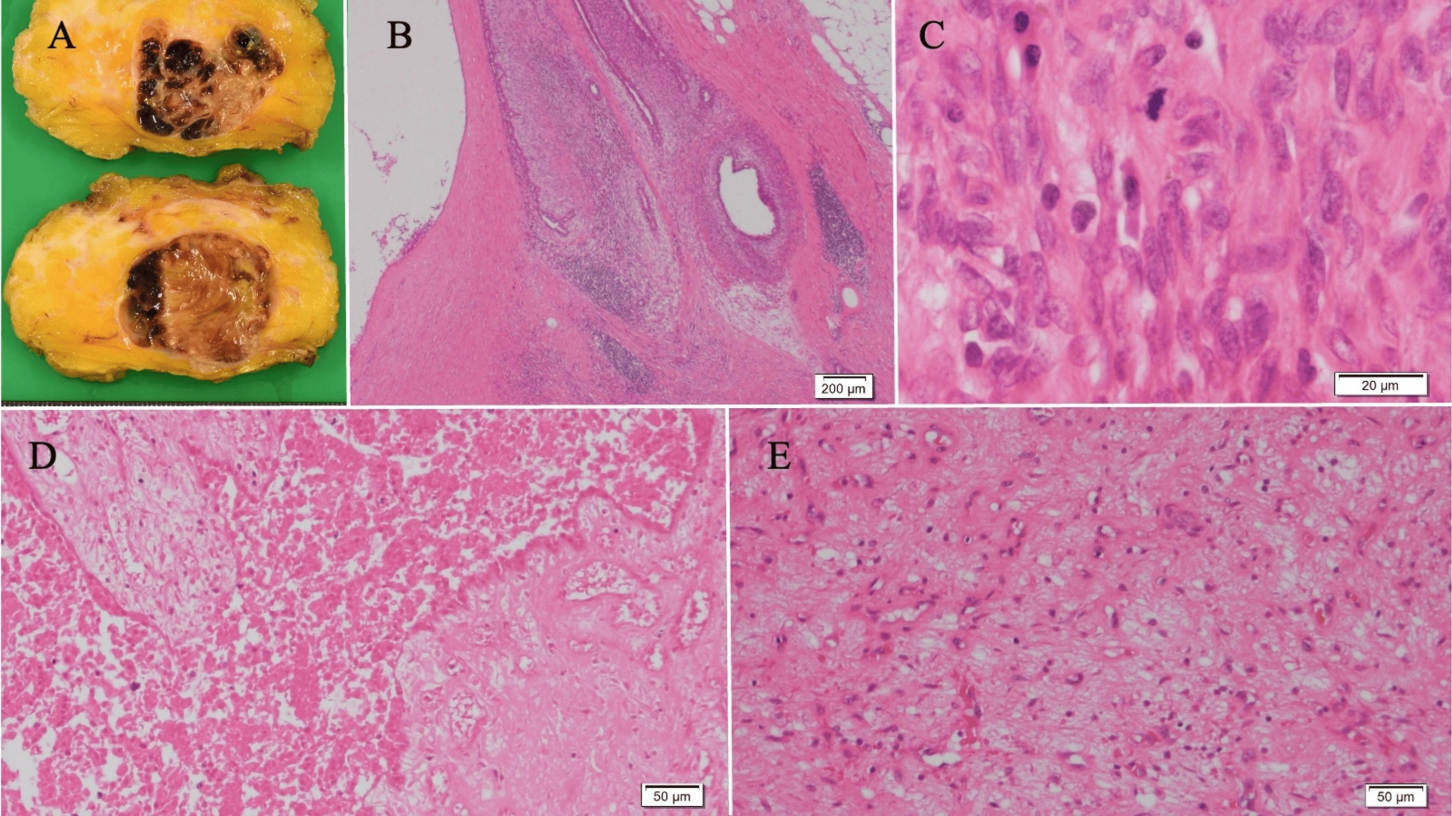
Pathological findings of the surgical specimen. (A) Gross appearance showing areas of hemorrhage. (B) Hematoxylin and eosin staining showed a leaf-like structure (scale bar: 200 μm). (C) The spindle cells showed mild cytopathic atypia and only a few mitotic figures (scale bar: 20 μm). (D) Infarction and (E) coagulation necrosis was observed (scale bar: 50 μm). CNB: Core needle biopsy

Since a part of the neoplastic lesion, such as intracystic carcinoma or malignant phyllodes tumor, could not be completely ruled out, an excisional biopsy was performed. Grossly, the tumor was a yellow nodular lesion with relatively clear borders and internal bleeding. The tumor had a lobular shape because of ductal epithelium with mild hyperplasia and hyperplasia of the interstitium centered on cells that looked like spindles. There were signs of damage inside the tumor, including a lot of coagulation necrosis and bleeding, groups of foamy histiocytes and hemosiderin-phagocytic histiocytes, and hyalinization, which was thought to be an infarction. There were some areas with high cell density, and most areas were affected by infarction and coagulation necrosis. The spindle cells showed mild cytopathic atypia and only a few mitotic figures. Based on these features, the pathological diagnosis was a borderline phyllodes tumor (Figure 4). The surgical margins were negative. The patient has shown no signs of recurrence for two years following surgery.

## Discussion

Phyllodes tumors are relatively rare mesenchymal tumors, as defined in the 1981 WHO histologic classification [1-3]. It is characterized by a lobulated, internal lobular (slit) structure. Secondary pathologic findings of hemorrhage, mucinous changes, gross necrosis and cystic degeneration may be present. Tumors are pathologically classified as benign, borderline, or malignant and require surgery due to their high proliferative potential.

Trauma, breastfeeding, pregnancy, and estrogen activity can facilitate the development of breast tumors. Infarct necrosis can lead to accelerated tumor development. Infarct necrosis may arise from several causes, including vascular blockage, thrombosis, vasculitis [4,5], biopsy puncture [6], relative ischemia necrosis, and tumor-specific immunological responses. The lower incidence of infarction in breast cancer compared to benign tumors is attributed to the malignant nature of cancer [7]. Breast cancers exhibit aggressive and abnormal cell proliferation, facilitating sufficient vascularization to meet their metabolic demands. In contrast, the comparatively limited angiogenesis increases the likelihood of infarction in benign breast tumors. These differences in the risk of infarction between cancerous and noncancerous breast tumors are caused by the complicated interactions between the tumor's features, its blood flow, and the immune system's response.

In this case, the pathological findings and images were compared in detail. The center of the phyllodes tumor exhibited mostly necrosis on contrast-enhanced CT scans and showed a high SI on T2-WI and a low SI on T1-WI with a low enhancement effect on MRI. The region close to the margin of the tumor observed necrotic liquefaction as a cystic area or necrotic cavity and showed a high SI both on T1-WI and T2-WI with a low enhancement effect. Similarly, areas with high SI on T1WI and T2WI, and significant enhancement were evaluated as areas with high tumor viability. This patient suddenly noticed a painful mass two years ago. The patient, a Filipino, had no history of precordial trauma and showed no obvious signs of thrombosis, but her diet was fatty and unbalanced. Based on the onset point and pathological findings, we assumed that hemorrhagic infarction had cut off the blood vessels supplying the tumor, causing necrosis. Blood flow returning (reperfusion) primarily weakens blood vessels in the brain, causing hemorrhagic infarction. Hemorrhagic infarction within a tumor, unlike cerebral infarction, occurs through a different mechanism related to tumor characteristics and growth rate [8]. Hemorrhage and infarction within the tumor lead to intratumoral necrosis.

Recent research has demonstrated that tumor necrosis influences metastasis-free survival in patients with neoplasms. Tumor necrosis is specifically associated with malignant phyllodes tumors [9]. The presence of necrosis or bleeding is also associated with local recurrence; hence, there are several reports that it is a factor in poor prognosis [10-12]. Intratumoral infarction may therefore be a confounding factor, as it is associated with independent prognostic factors such as tumor size, grade, hemorrhage, and necrosis. The most important factor related to local control is the margin at the time of surgical resection. In order to reduce local recurrence, it is important to perform surgical treatment with appropriate margins [13]. In particular, when necrosis or bleeding becomes extensive, diagnosis by needle biopsy may become difficult. To accurately identify potential diagnoses such as breast cancer or phyllodes tumors, it is crucial to perform a thorough analysis of both imaging findings and pathological images.

## Conclusions

We experienced a case of a phyllodes tumor that enlarged due to hemorrhagic infarction. Following a comprehensive diagnosis that included pathological findings and imaging tests, the patient underwent surgical resection with sufficient margins and is currently experiencing no recurrence. As in the current case, exact pathological diagnosis for the breast phyllodes tumors with severe hemorrhage and necrosis is challenging, but a comprehensive diagnosis that included imaging tests can recommend appropriate surgical treatment.
